# Comparison of radiomic feature aggregation methods for patients with multiple tumors

**DOI:** 10.1038/s41598-021-89114-6

**Published:** 2021-05-07

**Authors:** Enoch Chang, Marina Z. Joel, Hannah Y. Chang, Justin Du, Omaditya Khanna, Antonio Omuro, Veronica Chiang, Sanjay Aneja

**Affiliations:** 1 Department of Therapeutic Radiology, Yale School of Medicine, New Haven, USA; 2 Massachusetts Institute of Technology, Cambridge, USA; 3 Yale College, New Haven, USA; 4 Department of Neurosurgery, Thomas Jefferson University, Philadelphia, USA; 5 Yale Brain Tumor Center, New Haven, USA; 6 Department of Neurosurgery, Yale School of Medicine, New Haven, USA; 7 Center for Outcomes Research and Evaluation, Yale School of Medicine, New Haven, USA

**Keywords:** Cancer imaging, CNS cancer, Image processing, Machine learning

## Abstract

Radiomic feature analysis has been shown to be effective at analyzing diagnostic images to model cancer outcomes. It has not yet been established how to best combine radiomic features in cancer patients with multifocal tumors. As the number of patients with multifocal metastatic cancer continues to rise, there is a need for improving personalized patient-level prognosis to better inform treatment. We compared six mathematical methods of combining radiomic features of 3,596 tumors in 831 patients with multiple brain metastases and evaluated the performance of these aggregation methods using three survival models: a standard Cox proportional hazards model, a Cox proportional hazards model with LASSO regression, and a random survival forest. Across all three survival models, the weighted average of the largest three metastases had the highest concordance index (95% confidence interval) of 0.627 (0.595–0.661) for the Cox proportional hazards model, 0.628 (0.591–0.666) for the Cox proportional hazards model with LASSO regression, and 0.652 (0.565–0.727) for the random survival forest model. This finding was consistent when evaluating patients with different numbers of brain metastases and different tumor volumes. Radiomic features can be effectively combined to estimate patient-level outcomes in patients with multifocal brain metastases. Future studies are needed to confirm that the volume-weighted average of the largest three tumors is an effective method for combining radiomic features across other imaging modalities and tumor types.

## Introduction

Recent advances in machine learning and diagnostic imaging have increased enthusiasm surrounding the use of quantitative imaging to model clinical outcomes. One quantitative imaging technique that holds particular promise in modeling cancer outcomes is radiomic feature analysis. Radiomic features are quantitative metrics of size, shape, intensity, and texture that are extracted from medical images using high-throughput computational mining techniques^1,2^. These features represent unique radiographic signatures that have been shown to reveal prognostic insights about underlying gene-expression patterns^3^ and treatment response^4^ that may not be visible to the human eye.

Although radiomic features have shown the ability to risk stratify cancer patients^5,6^, radiomic analysis has largely been limited to the evaluation of individual tumor volumes. There is no established methodology regarding the best way to combine radiomic features for patients with multifocal sites of disease to establish a patient-level correlate of clinical outcomes. Within oncology, estimating patient outcomes among patients with multiple sites of metastatic disease is of particular interest as it influences multi-disciplinary treatment paradigms.

Prognostication for patients with metastatic disease is an area of increasing need, given as many as 49% of lung cancer patients within the United States are diagnosed with metastatic disease upon initial presentation^7^. More importantly, nearly 75% of patients with metastatic disease have greater than 5 lesions at diagnosis^8^. Even among non-metastatic cancer patients, as many as 17% of patients have multi-focal primary tumors^9^. Brain metastases represent a significant proportion of metastatic cancer patients, and conservatively, at least 50% of patients diagnosed with metastatic brain disease have multiple brain lesions^10^. With improved MRI brain imaging, it is likely that the number of patients found with multiple metastases is even higher^11^.

In this study, we attempted to identify optimal techniques to combine radiomic features for patients with multiple brain metastases to model patient-level outcomes. We compared various methods of combining radiomic features of individual tumors in patients with multiple brain metastases and evaluated the performance of different radiomic aggregation methods in estimating survival in patients using several different survival models.

## Results

### Patient demographics

A total of 831 patients with 3596 brain metastases were included. Patient demographics are presented in Table 1. Median overall survival was 12 months, median follow-up was 36 months, and 257 patients were right-censored. Median age was 63 years, and the most common primary malignancies were NSCLC (41.0%), melanoma (17.3%), breast (13.1%), SCLC (6.6%), renal (5.9%), and GI (5.3%). 543 patients (65.3%) had < 5 metastases, 214 (25.8%) had 5–10 metastases, and 74 (8.9%) had 11 + metastases.

**Table 1:** Patient demographics.

**Age**	
< 50	121 (14.6%)
50–59	199 (23.9%)
60–69	282 (33.9%)
> 70	229 (27.6%)
**Primary cancer site**	
NSCLC	341 (41.0%)
Melanoma	144 (17.3%)
Breast	109 (13.1%)
SCLC	55 (6.6%)
Renal	49 (5.9%)
GI	44 (5.3%)
Other	89 (10.7%)
**Number of metastases**	
< 5	543 (65.3%)
5–10	214 (25.8%)
11 +	74 (8.9%)
Karnofsky performance status*	
100	216 (29.1%)
90	161 (21.7%)
80	160 (21.6%)
70	102 (13.8%)
60	85 (11.5%)
50	13 (1.8%)
≤40	4 (0.5%)
**Presence of extracranial metastases**	
Yes	608 (73.2%)
No	223 (26.8%)

* If available.

### Overall results

Table 2 presents a comparison of various aggregation methods across several survival models.

**Table 2:** Comparison of aggregation methods.

Model	C-Index (95% CI)
**Cox proportional hazards**	
Unweighted average	0.610 (0.570–0.646)
Weighted average	0.604 (0.571–0.641)
Weighted average of largest 3 metastases	0.627 (0.595–0.661)
Largest + number of metastases	0.612 (0.579–0.649)
Largest metastasis	0.598 (0.559–0.636)
Smallest metastasis	0.595 (0.567–0.631)
**Cox proportional hazards with LASSO regression**	
Unweighted average	0.612 (0.585–0.647)
Weighted average	0.603 (0.573–0.640)
Weighted average of largest 3 metastases	0.628 (0.591–0.666)
Largest + number of metastases	0.597 (0.560–0.632)
Largest metastasis	0.596 (0.562–0.630)
Smallest metastasis	0.597 (0.557–0.630)
**Random survival forest**	
Unweighted average	0.649 (0.548–0.709)
Weighted average	0.641 (0.567–0.729)
Weighted average of largest 3 metastases	0.652 (0.565–0.727)
Largest + Number of metastases	0.622 (0.542–0.706)
Largest metastasis	0.627 (0.544–0.694)
Smallest metastasis	0.621 (0.529–0.709)

For the standard Cox proportional hazards model, the weighted average of the largest three metastases had the highest C-index (95% confidence interval) of 0.627 (0.595–0.661). The weighted average of the largest three metastases also had the highest C-index of all the aggregation methods on the Cox proportional hazards model with LASSO regression with a C-index of 0.628 (0.591–0.666) and on the random survival forest with a C-index of 0.652 (0.565–0.727).

### Sub-analysis: number of metastases

Table 3 presents model performance of sub-analyses based on number of brain metastases. For patients with < 5 metastases, the weighted average of their 3 largest metastases had the highest C-index on the standard Cox proportional hazards model with a C-index of 0.640 (0.600–0.686). For patients with 5–10 metastases, the unweighted average of all their metastases had the highest C-index of 0.697 (0.638–0.762). For patients with 11 + metastases, the model including only data from the largest metastasis as well as the number of metastases performed with the highest C-index of 0.909 (0.803–0.993).

**Table 3:** Sub-Analysis: Number of Metastases.

Model	C-Index (95% CI)
**< 5 Metastases (n = 543)**	
Unweighted average	0.621 (0.583–0.661)
Weighted average	0.617 (0.570–0.651)
Weighted average of largest 3 metastases	0.640 (0.600–0.686)
Largest + number of metastases	0.639 (0.593–0.676)
Largest metastasis	0.619 (0.580–0.653)
Smallest metastasis	0.612 (0.566–0.655)
**5–10 Metastases (n = 214)**	
Unweighted average	0.697 (0.638–0.762)
Weighted average	0.682 (0.617–0.743)
Weighted average of largest 3 metastases	0.688 (0.635–0.749)
Largest + number of metastases	0.691 (0.634–0.750)
Largest metastasis	0.688 (0.623–0.740)
Smallest metastasis	***
**11 + metastases (n = 74)**	
Unweighted average	0.876 (0.776–0.964)
Weighted average	0.872 (0.771–0.965)
Weighted average of largest 3 metastases	0.880 (0.787–0.964)
Largest + number of metastases	0.909 (0.803–0.993)
Largest metastasis	0.894 (0.765–0.974)
Smallest metastasis	***

*** Collinear.

### Sub-analysis: volume of largest metastasis

Table 4 describes model performance of sub-analyses based on volume of the largest metastasis. Across all sub-groups, the weighted average of the largest three metastases had the highest C-indices of 0.701 (0.652–0.748), 0.687 (0.622–0.728), 0.679 (0.623–0.733) for volumes < 0.200 cc, 0.200–0.700 cc, and > 0.700 + cc, respectively.

**Table 4:** Sub-analysis: volume of largest metastasis.

Model	C-Index (95% CI)
**Largest metastasis < 0.200 cc (n = 261)**	
Unweighted average	0.674 (0.630–0.729)
Weighted average	0.694 (0.639–0.749)
Weighted average of largest 3	0.701 (0.652–0.748)
Largest + number of metastases	0.698 (0.644–0.767)
Largest	0.695 (0.634–0.736)
Smallest	***
**Largest metastasis 0.200–0.700 cc (n = 281)**	
Unweighted Average	0.663 (0.615–0.719)
Weighted average	0.675 (0.622–0.731)
Weighted average of largest 3	0.687 (0.622–0.728)
Largest + number of metastases	0.668 (0.621–0.727)
Largest	0.656 (0.611–0.717)
Smallest	***
**Top 20 features largest metastasis 0.700 + cc (n = 289)**	
Unweighted average	0.671 (0.616–0.717)
Weighted average	0.656 (0.602–0.718)
Weighted average of largest 3	0.679 (0.623–0.733)
Largest + number of metastases	0.675 (0.614–0.715)
Largest	0.666 (0.615–0.714)
Smallest	***

***Collinear.

### Sensitivity analysis

Model performance was not improved when using the weighted average of the largest 2 and largest 4 metastases across survival models (Supplement 3). Model performance was lower when using PCA rather than mRMR for feature selection (Supplement 4). The Cox proportional hazards models with LASSO regression and random survival forest models using all 851 radiomic features did not have higher c-indices compared to models with prior feature selection – notably, the models using the weighted average of the largest three metastases continued to perform best (Supplement 5).

## Discussion

The emerging field of radiomic feature analysis has shown particular promise in cancer research. However, traditional radiomic feature analysis has had limited utility for patients with metastatic or multifocal disease because there is a paucity of established methods which aggregate radiomic features across multiple tumors to establish patient-level outcome estimates. Given the majority of cancer patients with brain metastases have multifocal disease, they represent an optimal patient population to study this question. We compared different methods of aggregating radiomic features and tested their performance across different survival models. Our study suggests that a volume-weighted average of the radiomic features of the largest three brain metastases is the most effective technique to model survival across various methods of survival analysis. Furthermore, this suggests that in patients with multi-focal disease, the largest tumors may be driving prognosis.

Practical implications of these findings include more efficient computational and clinical resource utilization. As server costs, data storage requirements, and computation time increase with increasing dataset size and complexity, effective models of prognosis may potentially be developed with just the data from the largest three metastases alone. Furthermore, clinicians may save time by only needing to manually segment the largest three metastases.

This study corroborates studies that have found radiomic features to correlate with prognosis among lung^12^, breast^13^, and prostate cancer patients^6^. To address the issue of combining multi-focal data at a patient-level, prior studies have implemented a weighted average of features of all metastases^14^ while others have included all tumors from a specific patient assigned to either a training or validation set to avoid cluster-correlation biases^15^. While tumor-level data may be useful for certain tasks like primary-site prediction, there is a need for aggregation of patient-level data for overall outcomes like survival or recurrence.

A notable finding of this study is that across all models, it was important that some measure of multi-focality was incorporated, whether aggregating radiomic features from the largest three metastases or including a clinical measure of multi-focality in the number of metastases.

On sub-analysis, across all volume groups of the largest metastasis, the weighted average of the largest three tumors had the highest performance. As the number of metastases increased greater than 5, models incorporating radiomic data from the largest metastasis with the addition of the number of metastases performed better, suggesting that with a higher number of metastases, additional non-imaging data (i.e. clinical data) may improve model performance, as prior studies have demonstrated the promise of clinical data alone in modeling survival^16^. Future studies are planned to incorporate both imaging and clinical data variables.

This study has limitations. First, there is an inherent selection bias since all the patients in this study were limited to those with brain metastases so results may vary across other disease sites. Second, these patients were all treated at one institution with the same MRI protocol. Efforts are underway to validate these findings at an independent institution. Third, these patients were ultimately treated with the same treatment modality. Further analysis is needed for patients treated with different interventions.

In conclusion, this study suggests that radiomic features can be effectively combined to establish patient-level outcomes in patients with multifocal disease. Future studies are needed to confirm that the volume-weighted average of the largest three tumors is an effective method for combining radiomic features across other imaging modalities and disease sites.

## Methods

### Patient data

Research was conducted in accordance with the Declaration of Helsinki guidelines and approved by the Yale University Institutional Review Board. Informed consent was obtained from all participants in this study. We analyzed 831 patients with 3,596 brain metastases treated with primary stereotactic radiosurgery (SRS) at our institution between 2000 and 2018. Patients with prior resection or prior radiation treatment were excluded. Metastases < 5 mm were also excluded. The primary outcome of interest was overall survival following SRS treatment defined as time from SRS treatment date to date of death or last follow-up.

### Image preprocessing

**Figure 1:**
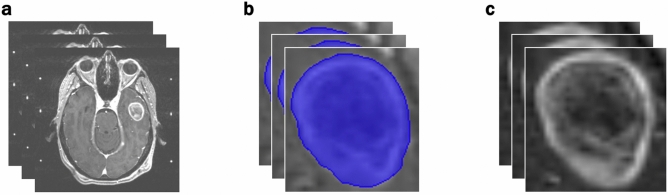
Preprocessing Workflow. (**a**) Input: slices of pre-treatment T1-post contrast brain MRI scans. (**b**) Identification of the region of interest from manual segmentations. (**c**) Output: extracted tumors with pixel resampling, N4ITK bias field correction, and z-score normalization.

The image preprocessing workflow is illustrated in Fig. 1. Individual metastases were segmented on T1-weighted contrast enhanced MRI images and approved by both a radiation oncologist and a neurosurgeon. A bounding box with 1 mm of peri-tumoral tissue around the maximum dimension of the tumor contour in the axial plane was created to ensure edge detection of the tumor. Images were resampled to 1 mm pixel spacing, corrected for low frequency intensity non-uniformity present in MRI data with the N4ITK bias field correction algorithm^17^, and z-score normalized to reduce inter-scan bias.

### Radiomic feature extraction and aggregation

851 radiomic features^2^ (Supplement 1) were extracted from the processed images of each brain metastasis and analyzed as predictors of survival. The following aggregation methods were compared to estimate patient-level risk for patients with multiple metastases: (1) Unweighted Average: Radiomic size and shape features were summed while all other features were averaged for each patient. (2) Weighted Average: Radiomic size and shape features were summed while all other features were averaged for each patient based on a weighted proportion of total volume of all metastases per patient^14^. (3) Weighted Average of Largest Three Metastases: Radiomic size and shape features from the largest three metastases of each patient were summed while all other features from the largest three metastases were averaged based on a weighted proportion of the total volume of the largest three metastases. The largest three metastases were chosen since the Graded Prognostic Assessment, a widely accepted model for prognostication, uses a cut-off of three metastases for prognostic risk stratification^16^. (4) Largest Metastasis + Number of Metastases: The features from the largest metastasis of each patient were selected, and the total number of metastases for each patient was included as an additional variable^16^. (5) Largest Metastasis Alone. (6) Smallest Metastasis Alone as a control with the assumption that the smallest tumor would have decreased prognostic value compared to larger tumors.

### Statistical analysis and survival models

The following survival models were tested: a traditional Cox proportional hazards model^18^, a Cox proportional hazards model with LASSO (least absolute shrinkage and selection operator) regression^19^, and a random survival forest^20^. Dimensionality reduction was performed at the patient level after radiomic feature aggregation. The top 20 ranked features (Supplement 2) were selected via minimum redundancy maximum relevance (mRMR)^21^, a method shown in multiple prior publications to effectively reduce dimensionality of radiomic features while maintaining the most relevant features for cancer prognostication^12,22,23^. Hyperparameter tuning for the Cox proportional hazards model with LASSO regression and the random survival forest model was performed with fivefold cross-validation using a grid search. Discriminatory ability of each model was assessed with calculated concordance indices (c-index) using 100 bootstrapped samples of 416 patients (50%). Multiple survival models were assessed to determine if particular aggregation methods were superior for specific survival models.

### Sub-group analysis

A sub-group analysis was performed to evaluate the role of the following potential drivers of patient prognosis: the overall number of metastases^16^ and the volume of the largest metastasis of each patient^24^. The robustness of the various radiomic aggregation techniques was evaluated by comparing the Cox proportional hazards model across these sub-groups: the number of metastases (< 5 metastases, 5–10 metastases, 11 + metastases) as well as the volume of the largest metastasis (< 0.200 cc, 0.200–0.700 cc, > 0.700 + cc) per patient.

### Sensitivity analysis

A sensitivity analysis was performed to compare the weighted average of the largest 2 and largest 4 metastases across survival models. Additionally, a sensitivity analysis was performed for the Cox proportional hazards models across aggregation methods using Principal Component Analysis (PCA) at the patient level post-aggregation to select a minimum number of features such that 90% of variance was retained. Finally, another analysis was performed where all 851 radiomic features were used for the Cox proportional hazards models with LASSO regression and the random survival forest models.

## Supplementary information

Supplementary Information.

## Data Availability

Radiomic features were extracted using PyRadiomics (version 2.2.0)^25^. Minimum redundancy maximum relevance feature selection was performed with the mRMRe package using R version 3.6.2^26^. Principal component analysis was performed with the scikit-learn package^27^. Image preprocessing and comparison of aggregation were performed with Python version 3.7. Survival analysis was performed with the lifelines and scikit-survival packages^28^. Code to reproduce our analysis is available at https://github.com/Aneja-Lab-Yale/Aneja-Lab-Public-BrainMetsRadiomics.
