# Supplementary material for: Comparison of radiomic feature aggregation methods for patients with multiple tumors
Source: Sci Rep. 2021 May 7;11:9758. doi: 10.1038/s41598-021-89114-6 (PMC8105371; doi:10.1038/s41598-021-89114-6)
Supplement: Supplementary file 1 — Supplementary Information. [file 41598_2021_89114_MOESM1_ESM.docx]

**SUPPLEMENTAL DATA**

**Comparison of Radiomic Feature Aggregation Methods for Patients with Multiple Tumors**

Enoch Chang^1^, Marina Z. Joel^1^, Hannah Y. Chang^2^, Justin Du^3^, Omaditya Khanna^4^,

Antonio Omuro^5^, Veronica Chiang^6^, Sanjay Aneja^1, 5, 7*^

1. Department of Therapeutic Radiology, Yale School of Medicine
2. Massachusetts Institute of Technology
3. Yale College
4. Department of Neurosurgery, Thomas Jefferson University
5. Yale Brain Tumor Program
6. Department of Neurosurgery, Yale School of Medicine
7. Center for Outcomes Research and Evaluation, Yale School of Medicine

**Contents:**

1. **Supplemental Table S1.** Radiomic Feature Overview
2. **Supplemental Table S2.** Top 20 Radiomic Features
3. **Supplement Table S3.** Sensitivity Analysis: Largest 2 and Largest 4 Metastases
4. **Supplement Table S4.** Sensitivity Analysis: Principal Component Analysis (PCA)
5. **Supplement Table S5.** Sensitivity Analysis: 851 Radiomic Features

**Supplemental S1.** Radiomic Feature Overview

The following Original feature classes were extracted using the Pyradiomics package.^1^ Features for each of the Wavelet filter classes (HHH, HLL, LHH, HHL, LHL, LLH, HLH, LLL) were also extracted for a total of 851 extracted radiomic features.

| **Original** | **Number of Features** | **Wavelet (HHH, HLL, LHH, HHL, LHL, LLH, HLH, LLL)** | **Number of Features** |
| --- | --- | --- | --- |
| Shape-Based (3D) | 14 |  |  |
| First Order Statistics | 18 | First Order Statistics | 18 |
| Gray Level Co-occurrence Matrix (GLCM) | 24 | Gray Level Co-occurrence Matrix (GLCM) | 24 |
| Gray Level Size Zone Matrix (GLSZM) | 16 | Gray Level Size Zone Matrix (GLSZM) | 16 |
| Gray Level Run Length Matrix (GLRLM) | 16 | Gray Level Run Length Matrix (GLRLM) | 16 |
| Neighboring Gray Tone Difference Matrix (NGTDM) | 5 | Neighboring Gray Tone Difference Matrix (NGTDM) | 5 |
| Gray Level Dependence Matrix (GLDM) | 14 | Gray Level Dependence Matrix (GLDM) | 14 |

**Supplemental Table S2.** Top 20 Radiomic Features

The top 20 radiomic features for each aggregation method based on mRMR feature selection.

| **mRMR Feature Importance Ranking** | **Unweighted Average** | **Weighted Average** |
| --- | --- | --- |
| 1 | shape LeastAxisLength | wavelet-LHH firstorder Maximum |
| 2 | wavelet-LLL glszm SizeZoneNonUniformityNormalized | shape MeshVolume |
| 3 | wavelet-HLH glszm SizeZoneNonUniformityNormalized | wavelet-HLL firstorder Entropy |
| 4 | wavelet-HHL firstorder RootMeanSquared | wavelet-HLH firstorder RootMeanSquared |
| 5 | wavelet-LHH firstorder Variance | wavelet-HLH glszm SizeZoneNonUniformityNormalized |
| 6 | wavelet-LLL glszm SmallAreaEmphasis | wavelet-LLH glszm GrayLevelVariance |
| 7 | wavelet-LHH firstorder RootMeanSquared | wavelet-HLH firstorder Variance |
| 8 | wavelet-LHH gldm SmallDependenceHighGrayLevelEmphasis | firstorder RootMeanSquared |
| 9 | firstorder RootMeanSquared | wavelet-HLL firstorder RootMeanSquared |
| 10 | wavelet-HLH glszm SmallAreaEmphasis | firstorder Variance |
| 11 | wavelet-HLH glszm LowGrayLevelZoneEmphasis | wavelet-LHH gldm SmallDependenceHighGrayLevelEmphasis |
| 12 | wavelet-HHL glszm GrayLevelNonUniformity | wavelet-HLH glszm SmallAreaEmphasis |
| 13 | wavelet-LLH glszm SmallAreaEmphasis | wavelet-LLH glrlm LongRunHighGrayLevelEmphasis |
| 14 | wavelet-LHL glszm SmallAreaEmphasis | wavelet-HHL firstorder RootMeanSquared |
| 15 | firstorder InterquartileRange | wavelet-HLL glszm SmallAreaLowGrayLevelEmphasis |
| 16 | wavelet-HLL firstorder RootMeanSquared | firstorder InterquartileRange |
| 17 | wavelet-LLL glszm LargeAreaLowGrayLevelEmphasis | wavelet-HLH glszm LowGrayLevelZoneEmphasis |
| 18 | gldm LowGrayLevelEmphasis | wavelet-LLL glszm SmallAreaEmphasis |
| 19 | wavelet-LHH glrlm LongRunHighGrayLevelEmphasis | wavelet-HLL glszm SmallAreaEmphasis |
| 20 | wavelet-HLL firstorder Entropy | wavelet-LHH glrlm LongRunHighGrayLevelEmphasis |

| **mRMR Feature Importance Ranking** | **Weighted Average of Largest 3 Metastases** | **Largest Metastasis** |
| --- | --- | --- |
| 1 | shape Elongation | wavelet-LHH firstorder Kurtosis |
| 2 | gldm LargeDependenceHighGrayLevelEmphasis | wavelet-LLH glszm GrayLevelVariance |
| 3 | wavelet-HHL firstorder 90Percentile | wavelet-LHL gldm SmallDependenceHighGrayLevelEmphasis |
| 4 | wavelet-LHH firstorder Variance | wavelet-HLH firstorder RootMeanSquared |
| 5 | wavelet-HLL glszm SmallAreaLowGrayLevelEmphasis | wavelet-HLH firstorder Variance |
| 6 | wavelet-HLH firstorder RootMeanSquared | wavelet-HLH glszm LowGrayLevelZoneEmphasis |
| 7 | firstorder RootMeanSquared | wavelet-LLL gldm LowGrayLevelEmphasis |
| 8 | firstorder Variance | wavelet-HLL glszm SmallAreaLowGrayLevelEmphasis |
| 9 | wavelet-LLH glszm GrayLevelVariance | wavelet-HLL firstorder RootMeanSquared |
| 10 | wavelet-LHL firstorder RootMeanSquared | wavelet-HHL firstorder RootMeanSquared |
| 11 | wavelet-HHL firstorder RootMeanSquared | wavelet-LLL glszm LargeAreaLowGrayLevelEmphasis |
| 12 | wavelet-LLH glszm GrayLevelNonUniformity | wavelet-HLL firstorder Entropy |
| 13 | wavelet-HLH glszm GrayLevelVariance | firstorder RootMeanSquared |
| 14 | wavelet-HLL firstorder RootMeanSquared | firstorder InterquartileRange |
| 15 | wavelet-LLH glszm SmallAreaEmphasis | wavelet-LLH glszm SmallAreaEmphasis |
| 16 | firstorder Maximum | wavelet-LHL glszm SmallAreaEmphasis |
| 17 | wavelet-LLL glszm SmallAreaEmphasis | wavelet-LHH glrlm LongRunHighGrayLevelEmphasis |
| 18 | wavelet-LHH glrlm LongRunHighGrayLevelEmphasis | wavelet-LHH glszm LowGrayLevelZoneEmphasis |
| 19 | wavelet-HLL glszm SmallAreaEmphasis | wavelet-LLL glszm SmallAreaEmphasis |
| 20 | wavelet-HLH glszm SizeZoneNonUniformityNormalized | wavelet-HHH firstorder RootMeanSquared |

| **mRMR Feature Importance Ranking** | **Smallest Metastasis** |
| --- | --- |
| 1 | wavelet-LHH glrlm LongRunEmphasis |
| 2 | wavelet-HHL firstorder RootMeanSquared |
| 3 | wavelet-HHH firstorder Variance |
| 4 | wavelet-LLL firstorder RootMeanSquared |
| 5 | wavelet-HLH glszm LargeAreaHighGrayLevelEmphasis |
| 6 | wavelet-LLL glszm SmallAreaEmphasis |
| 7 | wavelet-HLH glszm SmallAreaEmphasis |
| 8 | wavelet-HLH gldm SmallDependenceHighGrayLevelEmphasis |
| 9 | wavelet-HHH firstorder 90Percentile |
| 10 | wavelet-LLH glszm SmallAreaEmphasis |
| 11 | firstorder RootMeanSquared |
| 12 | wavelet-HHL glszm SmallAreaHighGrayLevelEmphasis |
| 13 | firstorder InterquartileRange |
| 14 | wavelet-LHL firstorder RootMeanSquared |
| 15 | wavelet-HHL firstorder Variance |
| 16 | wavelet-HLH firstorder Variance |
| 17 | wavelet-LLL glszm SizeZoneNonUniformity |
| 18 | wavelet-HHH glrlm LongRunHighGrayLevelEmphasis |
| 19 | wavelet-LLL glszm GrayLevelNonUniformity |
| 20 | wavelet-HLL glszm SmallAreaEmphasis |

**Supplement Table S3.** Sensitivity Analysis: Weighted Average of Largest 2 and Largest 4 Metastases

The model performance for the weighted average of the largest 2 and largest 4 metastases was compared across the Cox proportional hazards model, Cox proportional hazards model with LASSO regression, and Random Survival Forest model.

| **Weighted Average of Largest 2 Metastases** | |
| --- | --- |
|  | **C-Index (95% CI)** |
| Cox Proportional Hazards | 0.592 (0.554-0.629) |
| Cox Proportional Hazards with LASSO Regression | 0.624 (0.596-0.657) |
| Random Survival Forest | 0.647 (0.567-0.706) |
|  |  |
| **Weighted Average of Largest 4 Metastases** | |
|  | **C-Index (95% CI)** |
| Cox Proportional Hazards | 0.623 (0.595-0.668) |
| Cox Proportional Hazards with LASSO Regression | 0.627 (0.590-0.664) |
| Random Survival Forest | 0.646 (0.557-0.713) |

**Supplement Table S4.** Sensitivity Analysis: Principal Component Analysis (PCA)

PCA feature selection was performed at the patient level post-aggregation for the Cox proportional hazards model. The number of components were selected to retain 90% variance.

| **Cox Proportional Hazards (PCA Selection)** | |
| --- | --- |
| **Model** | **C-Index (95% CI)** |
| Unweighted Average | 0.602 (0.568-0.628) |
| Weighted Average | 0.612 (0.590-0.636) |
| Weighted Average of Largest 3 Metastases | 0.620 (0.596-0.642) |
| Largest + Number of Metastases | 0.611 (0.594-0.639) |
| Largest Metastasis | 0.617 (0.601-0.622) |
| Smallest Metastasis | 0.596 (0.570-0.628) |

**Supplement Table S5.** Sensitivity Analysis: 851 Radiomic Features

The model performance was compared using all radiomic features for the Cox proportional hazards models with LASSO regression as the random survival forest models.

| **Cox Proportional Hazards with LASSO Regression (851 Features)** | |
| --- | --- |
| **Model** | **C-Index (95% CI)** |
| Unweighted Average | 0.557 (0.466-0.615) |
| Weighted Average | 0.564 (0.489-0.612) |
| Weighted Average of Largest 3 Metastases | 0.571 (0.488-0.612) |
| Largest + Number of Metastases | 0.566 (0.527-0.605) |
| Largest Metastasis | 0.563 (0.487-0.610) |
| Smallest Metastasis | 0.555 (0.499-0.596) |
|  |  |
| **Random Survival Forest (851 Features)** | |
| **Model** | **C-Index (95% CI)** |
| Unweighted Average | 0.609 (0.553-0.662) |
| Weighted Average | 0.609 (0.539-0.664) |
| Weighted Average of Largest 3 Metastases | 0.611 (0.545-0.668) |
| Largest + Number of Metastases | 0.605 (0.535-0.668) |
| Largest Metastasis | 0.607 (0.567-0.657) |
| Smallest Metastasis | 0.602 (0.533-0.662) |

**References**

1 van Griethuysen, J. J. M. *et al.* Computational Radiomics System to Decode the Radiographic Phenotype. *Cancer Res* **77**, e104-e107, doi:10.1158/0008-5472.CAN-17-0339 (2017).
